# Modelling and Optimization of Polycaprolactone Ultrafine-Fibres Electrospinning Process Using Response Surface Methodology

**DOI:** 10.3390/ma11030441

**Published:** 2018-03-17

**Authors:** Adhi Anindyajati, Philip Boughton, Andrew J. Ruys

**Affiliations:** School of Aerospace, Mechanical and Mechatronic Engineering, The University of Sydney, NSW 2006, Australia; philip.boughton@sydney.edu.au (P.B.); andrew.ruys@sydney.edu.au (A.J.R.)

**Keywords:** electrospinning, polycaprolactone, response surface methodology, fibre diameter, elastic modulus

## Abstract

Electrospun fibres have gained broad interest in biomedical applications, including tissue engineering scaffolds, due to their potential in mimicking extracellular matrix and producing structures favourable for cell and tissue growth. The development of scaffolds often involves multivariate production parameters and multiple output characteristics to define product quality. In this study on electrospinning of polycaprolactone (PCL), response surface methodology (RSM) was applied to investigate the determining parameters and find optimal settings to achieve the desired properties of fibrous scaffold for acetabular labrum implant. The results showed that solution concentration influenced fibre diameter, while elastic modulus was determined by solution concentration, flow rate, temperature, collector rotation speed, and interaction between concentration and temperature. Relationships between these variables and outputs were modelled, followed by an optimization procedure. Using the optimized setting (solution concentration of 10% *w*/*v*, flow rate of 4.5 mL/h, temperature of 45 °C, and collector rotation speed of 1500 RPM), a target elastic modulus of 25 MPa could be achieved at a minimum possible fibre diameter (1.39 ± 0.20 µm). This work demonstrated that multivariate factors of production parameters and multiple responses can be investigated, modelled, and optimized using RSM.

## 1. Introduction

Polymeric fibres have been widely explored as scaffolds in tissue engineering applications and regenerative medicine. They offer the potential to mimic the architecture of extracellular matrix (ECM), which comprises a fibrous network [1,2]. A scaffold ideally satisfies requirements including a porous structure with interconnected pores suitable for cell growth and exchange of nutrients, biocompatibility, and mechanical properties similar to the substituted tissue [3,4,5]. Furthermore, scaffold structure dictates cell growth and matrix deposition for tissue formation [6,7,8,9]. Therefore, it is important for a scaffold design to mimic the structure and properties of the native tissue.

Ultra-fine fibres can be fabricated using electrospinning, which is versatile and efficient for producing non-woven networks with fibre diameters ranging from 3 nm to 5 µm [10]. This technique has been broadly explored for numerous engineering purposes, including filtration, protective clothing, optical electronics, solar sails, light sails, mirrors for use in space, pesticides, reinforced composites, and biomedical devices [10,11,12,13]. Wound dressings, drug delivery systems, tissue engineering, and structural elements in artificial organs are some examples of electrospun fibres in medical applications. Using electrospinning, fibrous structures can be tailored to possess unique characteristics, such as high surface area, extreme length up to kilometres in magnitude, and alignment on a molecular level [14]. Hence, the development of fibrous scaffolds for tissue engineering could benefit from the electrospinning technique.

Recent studies on electrospinning for tissue engineering application included the use of electrospun fibres combined with other materials. Composite of hydrogel/electrospun collagen scaffold was fabricated to mimic native extracellular matrix for meniscus tissue regeneration [15]. Electrospun meshes of poly(lactic-co-glycolic acid) and nanohydroxyapatite were modified using poly(allylamine hydrochloride) and poly(sodium 4-styrenesulfonate) as polyelectrolytes through layer-by-layer assembly, followed by peptides incorporation, to stimulate bone healing [16]. Layer-by-layer coating of chitosan and collagen was also introduced onto co-electrospun PCL/cellulose acetate nanofibrous matrix for wound healing application [17]. Alternating process of PCL electrospinning and inkjet printing of chondrocytes suspended in a fibrin–collagen hydrogel was developed to fabricate a construct for cartilage tissue engineering [18].

In the process of product development, including that of biomedical products and devices, quality by design and manufacturing process takes an important role. This approach was recommended by the FDA (Food and Drug Administration) and ISO 13485 (international standard of quality management systems for medical devices) [19,20]. Quality is defined as product ability to meet customer satisfaction and these requirements of quality need to be translated into specification [21]. Quality characteristics of a product are dependent on multivariate processing variables. For this, a designed experiment offers a strategy to simultaneously investigate multiple variables defining process or product quality. This approach is obviously more efficient compared to a one-factor-at-a-time strategy [22]. There are several techniques in the design of experiments, including factorial, robust parameter design (Taguchi’s method), and the response surface method (RSM) [22]. This study will be focused on the latter.

Response surface methodology is a collection of statistical design and numerical optimization techniques used to optimize a process and product design, and has become the core of industrial experimentation [23]. Gaining advantage from computer technology and software development, RSM provides techniques for reducing variance and for process improvement [24]. RSM offers potential benefits compared to other optimization techniques, for example shorter computational modelling and ability to suggest straightforward optimization with comparably high accuracy [25]. Using this method, which mostly is using either first-order or second-order polynomial models for function estimation, an empirical relationship between independent variables and one or more response variables is obtained [26]. Users of the RSM approach have broadened, from chemicals, foods, and manufacturing, to biological, biomedical, and biopharmaceutical. It has even been considered as the standard for optimization experiments, both in laboratory and industrial settings [27]. The application of RSM on process and product optimization has been reported in literature. RSM was used with multicriteria decision analysis to optimize the production of vancomycin nanoparticles to achieve desired particle size and encapsulation efficiency [27]. On optical fibre coating, RSM was utilized to build prediction model and subsequently optimize the process, resulting in the improved contraction rate of the outer coating [28]. In the area of biotechnology, RSM was applied to optimize product formulation and operating conditions in many cases, for example to find the important components for cells medium and their optimum amount to improve microbial transglutaminase (MTGase) activity [19].

In this study, a scaffold for acetabular labrum implant was developed from electrospun polycaprolactone (PCL) fibres and the properties emphasized were mechanical properties and fibre diameter. This device is proposed to aid recovery in labral injury, as well as an alternative treatment for labral reconstruction. Electrospinning is a process that involves multivariate factors determining the properties of resultant fibres. In general, they can be grouped into three parameters: solution properties, processing variables, and ambient parameters [12,29]. Solution properties include viscosity, conductivity, surface tension, molecular weight of the polymer, and dielectric constant. Control factors comprise flow rate, electric field, tip to collector distance, needle tip design, and collector material and geometry. Ambient parameters, such as temperature, humidity, and air velocity may also influence the result. Combinations of these factors determine fibre diameter, uniformity (beading formation), and alignment [7,12,30,31,32,33]. Simultaneously, mechanical properties will also be affected.

In investigating electrospun fibre properties with respect to the multivariate nature of electrospinning, the RSM approach has been applied in numerous electrospinning studies of various materials [26,32,34,35,36,37,38,39,40,41]. These investigations demonstrated that this technique could develop a predictive model along with confirmation of validity. Furthermore, optimum settings to achieve the desired output or response can be obtained [34,37]. Mostly, fibre diameter was the response under investigation. Although these studies were conducted using different materials and experimental conditions, they appeared to be in good agreement, in which the most influential factors for fibre diameter are material content or concentration [26,32,34,36,37,38,39,40] and voltage [26,32,34,35,36,37,40]. Only a few studies examined the effect on mechanical properties. Optimization was also developed only for a single response. When different materials and experimental conditions are involved, the results obtained are usually specific to the case under study. For different cases, particular designs of experiments need to be developed.

This present study explored the use of RSM in PCL electrospinning to investigate the effect of parameter settings on fibres properties. Parameters under investigation were solution concentration, flow rate, collector to needle distance, solution temperature, and mandrel rotation speed. Furthermore, this paper will report the use of RSM to find optimum parameter setting combinations to simultaneously achieve desired multiple responses. To the author’s knowledge, there are currently no literature reports on optimization of electrospinning parameter for multiple responses. Two responses representing quality of fibrous scaffolds were examined: fibre diameter and elastic modulus. Optimization then followed to achieve the desired quality, which was defined by minimum fibre diameter and an elastic modulus of 25 MPa. The smallest diameter is preferred since it can provide a higher surface area, allowing more binding sites for cell adhesion [42]. A target value of 25 MPa for elastic modulus was preferred so as to match the mechanical properties of human acetabular labrum [43].

## 2. Results

### 2.1. Experimental Runs

Electrospinning parameters and their levels, in coded and real value, are listed in Table 1. The levels of the five factors were chosen based on the minimum and maximum limitation of the system being used. Thirty two experimental runs were conducted based on CCD (central composite design) suggested by Minitab 17 software (Minitab Inc., State College, PA, US). The experimental conditions and their responses are summarized in Table 2. Data obtained from average fibre diameter and Young’s modulus were then analysed using Minitab 17, in order to investigate the relationship between experimental conditions and the response.

### 2.2. Fibre Diameter

Minitab software provided analysis for the experiment datasheet of the response surface design. The outputs included *p*-value (significance), R^2^ (ability to explain variance), and unusual observation (outlier). The results of response surface analysis for fibre diameter are summarized in Table 3. The response surface regression suggested that the model adequately fits the data (*p* > 0.05 for lack-of fit). However, adjusted R^2^ (9.06%) and predicted R^2^ (0.00%) are low and not in reasonable proximity. The only significant value is concentration (*p* < 0.05). Consequently, the generated model needs to be improved by removing insignificant factors and influential outliers. After two iteration steps, a final model was obtained. Lack of fit calculation (*p* > 0.05) suggested that the model still fits the data. Adjusted R^2^ and predicted R^2^ were also improved, showing 37.02% and 31.77%, respectively. It suggested that the model could have more coverage in variance data, as well as better accuracy. The mathematical model obtained from the final model is presented in Equation (1):

diameter = −0.771 + 0.2450A.(1)

Despite this improvement, both the adjusted and predicted R^2^ are still relatively low. Nevertheless, the important finding was that this study revealed that the most important factor influencing fiber diameter obtained from the electrospinning system was concentration.

**Figure 1:**
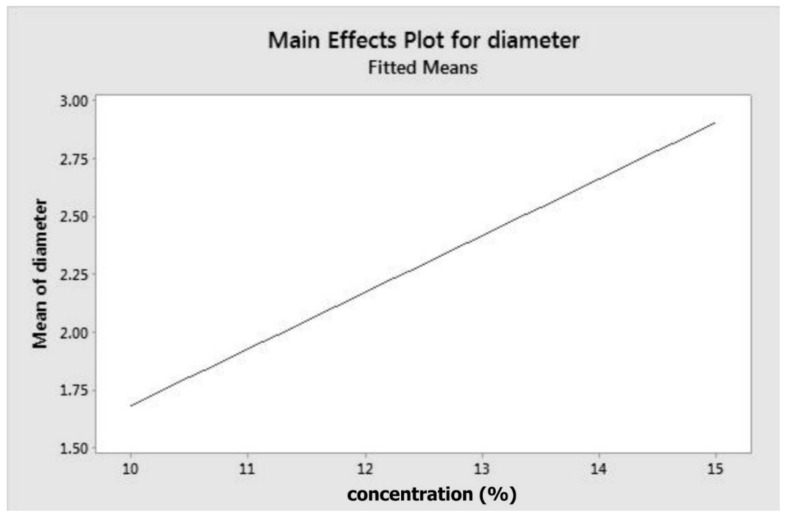
Main effect plot for fibre diameter.

The model (Equation (1) and main effect plot (Figure 1)) indicate that concentration has a positive correlation with fibre diameter. To obtain fibres with smaller diameters, a lower concentration is required, as depicted by Figure 1. At minimum concentration, which is of 10% *w*/*v*, fibre diameter is around 1.7 µm. The equation or model proposed is only valid for the experimental conditions applied in this investigation and would need to be regenerated or refined for any addition or modification of the parameters.

**Figure 2:**
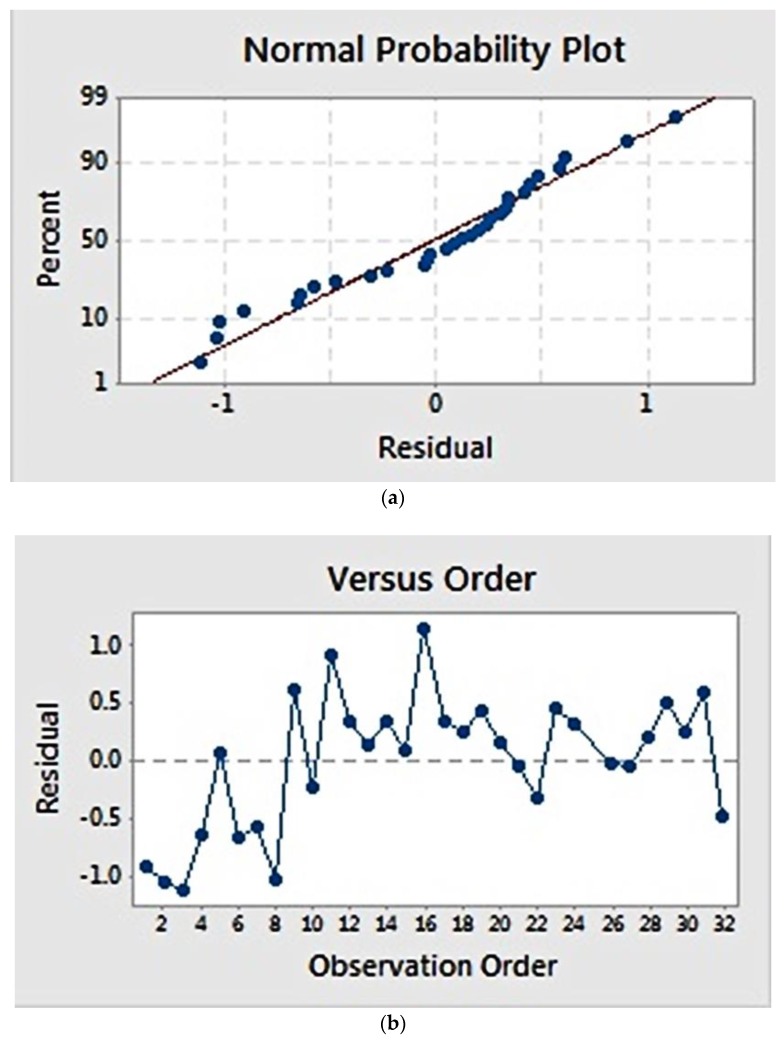
Residual plot for fibre diameter: (**a**) normal probability plot, showing a linear correlation; (**b**) residuals versus observation order, showing a random correlation.

Residual plots (Figure 2) provide visual examination about the model validity. Residual is defined as the difference between the observed and estimated value [22]. Normal probability plots (Figure 2a) visualize the residuals with respect to the expected value when the distribution is normal. The graph showed that the residuals appear to correlate linearly, suggesting that the errors are normally distributed. Additionally, a graph of residuals vs. observation order (Figure 2b) examines whether the residuals are independent of the order of data observation. Residuals on the graph generally showed a random pattern, suggesting that the observation sequence did not influence the result and that there was therefore no correlation among the residuals.

### 2.3. Elastic Modulus

Table 4 summarizes the results of response surface analysis for elastic modulus. This analysis showed a lack of fit value of 0.855, indicating that the quadratic model fits the data. Several factors were considered significant in determining elastic modulus (*p* < 0.05), including concentration, flow-rate, temperature, and rotation. Some interactions were also influential (*p* < 0.05), which were concentration–temperature and distance–temperature. The fitted model possessed high adjusted R^2^ (81.18%), suggesting that the model was able to explain variance in the data. However, the predicted R^2^ was low (0.00%), indicating that the model may not be able to predict the response of new observations well. To improve the model, several iterations were carried out by removing insignificant terms and influential outliers. After the model was refined, predicted R^2^ was improved (73.24%) and in considerably firm agreement with adjusted R^2^ (78.20%). These terms indicate that the model had substantial coverage in variance data, as well as better accuracy. The mathematical model obtained from the final model is as follows:

Elastic Modulus = −296.1 + 25.71A + 8.84B + 5.59D + 0.01809E − 0.566AD.(2)

**Figure 3:**
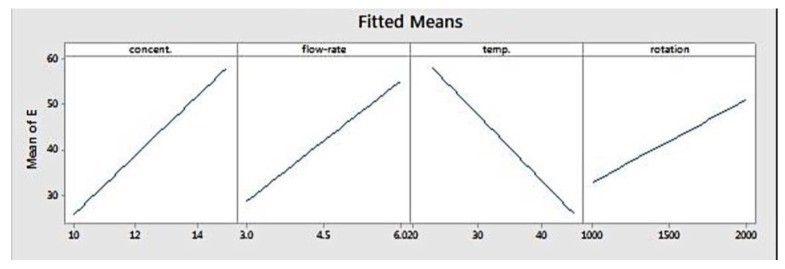
Main effects plot for modulus elasticity.

**Figure 4:**
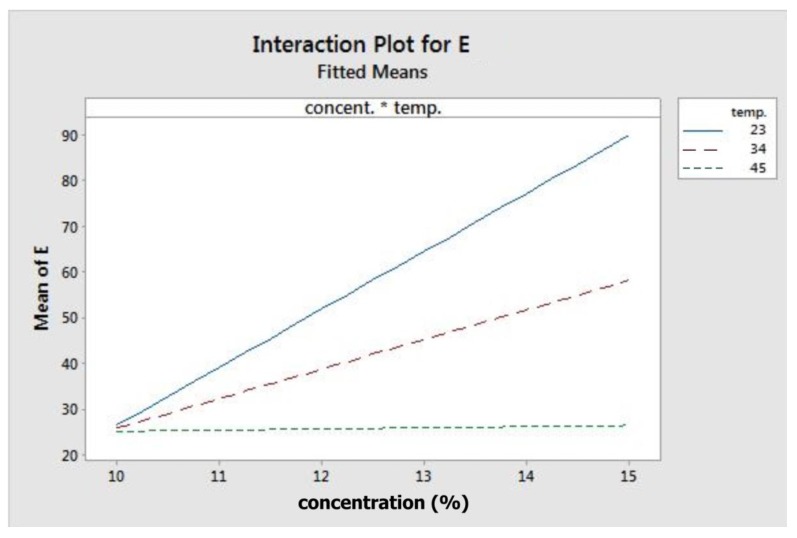
Interaction plot for modulus elasticity.

Figure 3 depicts the fitted means of modulus of elasticity as a function of concentration, flow rate, temperature, and rotation. Based on that figure, concentration, flow rate, and rotation had a positive correlation with elastic modulus, while temperature showed the opposite. This means that higher level settings of concentration, flow rate, and rotation will yield fibres with a higher elastic modulus. On the other hand, increasing temperature will decrease fibre elastic modulus. Figure 4 shows the interaction effect of concentration and temperature on modulus of elasticity. The effect of concentration on fibre elastic modulus depends on temperature. At the highest temperature, elastic modulus was relatively similar across all concentration levels. At the middle temperature range, increasing concentration improved the elastic modulus. This improvement was more pronounced when the system temperature was set at the lowest level. According to the graph, the highest modulus can be achieved when concentration is set at the highest level and at the minimum temperature.

**Figure 5:**
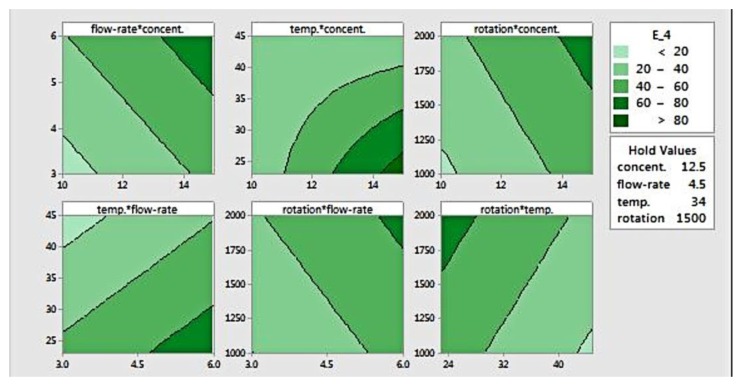
Contour plot for modulus elasticity.

The response surface can be visualized using contour plots, as presented in Figure 5, which is useful for defining desired response and operating parameters. This plot defines the relation between a response and two variables, based on a model equation. For this elastic modulus data, the contour plot shows paired factors plotted in the *x*- and *y*-axis, while the other factors are held constant. The contour areas denote constant responses related to elastic modulus of 20, 40, 60, and 80 MPa. Areas with the darkest colour indicate a location where the stiffness is the highest (>80 MPa).

The contour plots for flow rate versus concentration, rotation versus concentration, and rotation versus flow-rate show that increasing those three factors (flow rate, concentration, and rotation) results in higher elastic modulus. Setting those parameters at the highest level will yield fibres with the highest modulus. As for temperature, when it is plotted against both flow rate and rotation, it was found that that a higher temperature produced fibres with lower stiffness. Higher modulus could be achieved when temperature was set at its lowest level, combined with the highest level of flow rate and/or rotation.

The plot of temperature versus concentration shows curvature, indicating that there is interaction between both factors. That is, the effect of either temperature or concentration is not always the same. At lower concentration, temperature doesn’t have an effect on fibre modulus, as opposed to a higher concentration setting. Conversely, concentration shows a clear effect on elastic modulus at lower temperatures. This graph also shows that the elastic modulus increases as the concentration rises and the temperature reduces simultaneously. Additionally, it suggests that the elastic modulus can be maximized at a concentration of 15% *w*/*v* and a temperature of 25 °C or below.

**Figure 6:**
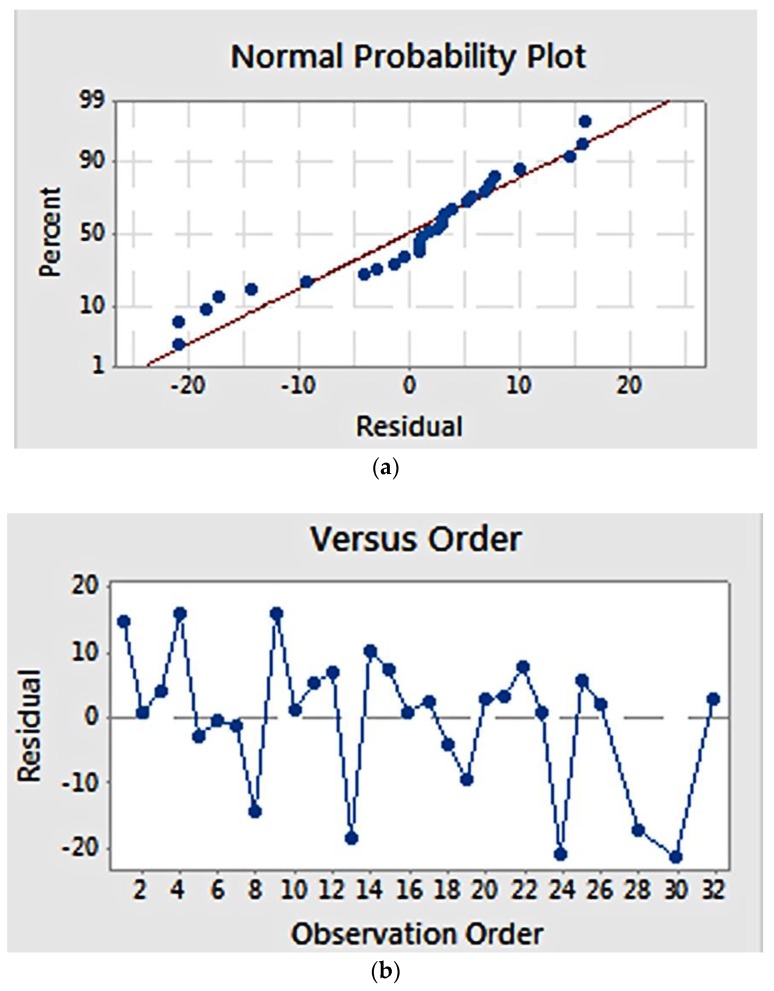
Residual plot for elastic modulus: (**a**) normal probability plot, showing a linear correlation; (**b**) residuals versus order, showing a random correlation.

Validity of the model estimation was examined using a normal probability plot and residual versus observation order plot (Figure 6). The normal probability plot (Figure 6a) showed that the residuals pattern followed a straight line, suggesting a normal distribution of residuals. Meanwhile, the plot of residuals versus order (Figure 6b) suggests that estimation errors were not related to the order of observation, as the residuals showed a random pattern

### 2.4. Parameter Optimization and Confirmation Test

In this experiment, the design parameter for response optimization is set to obtain a minimum fibre diameter and a modulus of elasticity of 25 MPa. The goal for diameter is to minimize it. Since the optimizer procedure required boundary values to be set, a value of 120 nm was considered as a target and values above 2 µm were unacceptable. For elasticity, the goal was to obtain a value at or near 25 MPa, where values less than 14 MPa or greater than 36 MPa were unacceptable. These values are based on the properties of acetabular labrum that possessed a fibril diameter of 120 nm and a tensile modulus of 24.7 ± 10.8 MPa [43,44]. This optimization was run based on the regression model suggested by response surface analysis.

**Figure 7:**
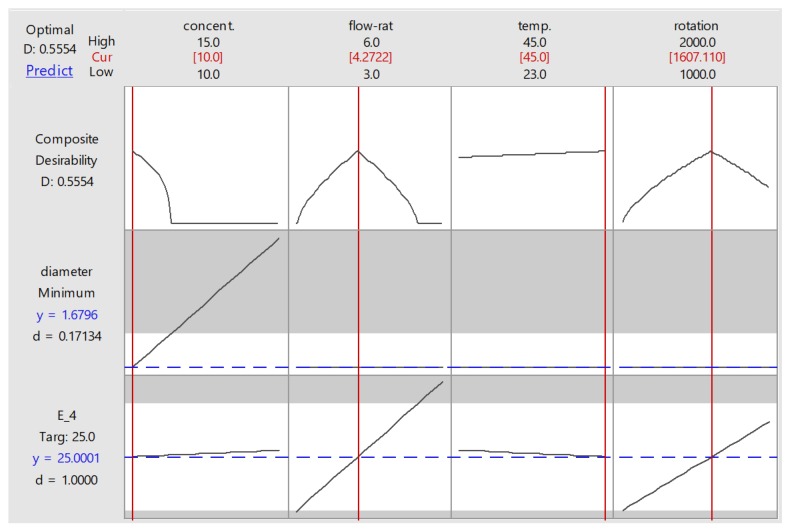
Optimization plot provided by response optimizer.

Figure 7 shows the optimized parameter setting suggested by the response optimizer. To obtain a modulus elasticity of 25 MPa at minimum possible fibre diameter (1.68 µm), the suggested levels for concentration, flow rate, temperature, and rotation were 10% *w*/*v*, 4.27 mL/h, 45 °C, and 1610 RPM. Needle–collector distance was not involved, since it was considered an insignificant factor. This graph also presents the desirability factor (D), representing how close the response obtained from a particular setting was to the goal. Each response has its individual desirability, which will be combined into composite desirability of multiple responses being optimized. In this study, apparently the individual desirability of fibre diameter was very low (0.17) because the minimum value set at 120 nm was nearly impossible to obtain. It also affected composite desirability, which only reached 0.555. The desirability factor was also influenced by response weighting. In this study, the weights for both diameter and elastic modulus were set at 1, representing equal importance. If the weight for modulus was set higher than that of diameter, the composite desirability would increase since modulus had a higher individual desirability value.

Independent tests were then conducted to confirm the suggested optimized setting. For practicality, the test was set at 10% *w*/*v*, 4.5 mL/h, 45 °C, and 1500 RPM. The optimizer predicted that the results for both diameter and modulus from this setting will be 1.68 ± 0.37 µm and 25.1 ± 10.7 MPa at the 95% confidence interval. Nine runs were conducted, varied by days (day 1, 2, and 3) and distance (10, 12.5, 15 cm). The samples were then tested using protocols similar to the optimization runs. The results are summarized in Table 5.

**Figure 8:**
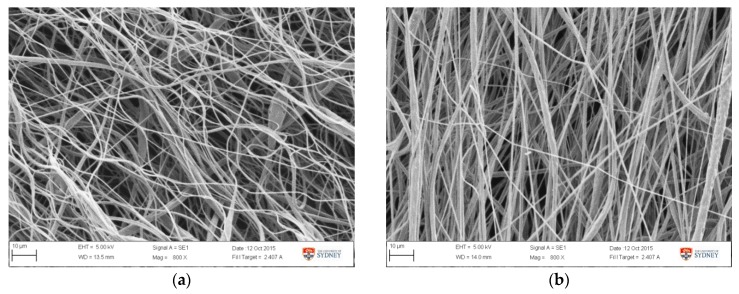
SEM image of fibre morphology obtained from the optimized setting from (**a**) day 1, run 2 and (**b**) day 2, run 6.

The validation result (Table 5) showed that the average fibre diameter obtained was 1.39 ± 0.20 µm, which is in the lower range of the predicted 1.68 ± 0.37 µm. This is attributed to the lower diameter value obtained from earlier runs, as showed in Table 5 and Figure 8. Samples from earlier runs have bead and string morphology with smaller fibre diameter (Figure 8a), while the other group showed more uniform and thicker fibres (Figure 8b). Variance in validation results is likely attributed to the non-uniform morphology observed in the early runs, as it was also observed in the samples obtained from experimental tests (Appendix A1). SEM images from all validation runs are also attached in Appendix A2. The formation of beads and string morphology in the early runs in both of experimental and validation runs was possibly due to a viscosity factor. In the earlier runs, it was possible that the viscosity was slightly lower, due to incomplete PCL/acetone dissolution prior to electrospinning. If data from these early-run beaded fibres are eliminated, the diameter becomes 1.51 ± 0.10 µm, which is close to the predicted value of 1.68 ± 0.37 µm. Compared to the fibre resulting from experimental runs, these optimized fibres possessed smaller diameters, improved uniformity, and were relatively bead-free.

For elastic modulus, confirmation tests resulted in fibres with an average modulus of 26.0 ± 8.6 MPa, which is in firm agreement with the predicted 25.1 ± 10.7 MPa. This test also suggests that elastic modulus was more predictable with respect to variation than fibre diameter. It also appears to not be correlated to fibre beading and uniformity, although several studies suggested that fibre diameter may affect elastic properties [45,46,47]. Despite variation in fibre morphology, the stiffness across samples is relatively identical.

## 3. Discussion

The relationship between a series of electrospinning parameters and their outcomes has been investigated and modelled using RSM, along with factors that impact output characteristics. For fibre diameter, solution concentration was the only significant factor. Secondly, solution concentration, flow rate, temperature, collector rotation speed, and interaction between concentration and temperature were the influential factors for the fibre elastic modulus. The fibre diameter model showed relatively low predicted R^2^ (31.77%), suggesting that it should be exploited more carefully for predicting future data, by considering possible sources of variance. However, the predicted R^2^ (78.20%) for fibre stiffness was reasonably high and could be used more confidently.

Solution concentration and fibre diameter appeared to have a positive correlation. The increase in fibre thickness following the increasing concentration was documented in the literature, both in electrospun PCL and other polymers [26,30,32,34,38,39,40,41,45,48,49,50,51,52,53]. It proved to be one of the most influential factors regarding fibre diameter [34]. Higher solution concentration led to more significant chain entanglement [32]. Consequently, it possessed higher viscoelasticity to resist elongation from electrostatic forces. At high concentration, viscoelastic force overcame surface tension, contributing to thicker fibre formation but less beading or droplets [41,48,54]. The relation between concentration and fibre diameter could be either quadratic or linear [41,49]. Additionally, there was also a certain concentration limit above which fibres could not be formed at all [51,53].

Regarding mechanical properties, the positive impact of solution concentration was also confirmed by several studies [26,35,39]. The higher modulus obtained was associated with the aligned polymer chain along the fibre axis [26]. Polymer solutions with lower concentration might lack effective chain entanglements, which are required for the extension of main chain segments without slippage and molecular relaxation effects [55]. Furthermore, mechanical properties of polymer and electrospun polymer fibres were related to chain ordering or crystallinity [56,57,58,59].

Investigations of temperature effect on elastic modulus appear to be rarely reported compared to those on fibre morphology. The elevation of temperature generally reduced fibre diameter, through molecule expansion, reduction in chain entanglements, viscosity, and surface tension, as well as increased elongation [12,60,61,62,63,64,65]. High thermal application could also decrease fibre crystallinity by accelerating solvent evaporation, thus prohibiting chain ordering [62]. As mechanical properties were correlated to chain entanglement and alignment, it is possible that raising temperature can reduce fibre modulus of elasticity [26,55].

In PCL electrospinning, temperature showed interdependency with concentration on molecular orientation. It was reported that, in all concentrations, molecular alignment was improved from a temperature of 25 to 35 °C, but was then decreased after further increase to 40 °C. Increased temperature helped chain relaxation, thus reducing molecular orientation and crystallinity [64]. In another study, the drawing process of gravity-spun PCL fibre at room temperature involved breakdown and unfolding of crystalline units, as well as extension of amorphous segments [55]. This extension was eased by the low glass transition temperature (T_g_) of PCL, which enables high chain mobility at room temperature. This probably explains why, in this present study, concentration had almost no effect on fibre elasticity at the higher temperature (45 °C), as the chain was already relaxed, thus there was no increase in crystallinity. In electrospinning at room temperature, the chain mobility was probably already facilitated along with slower solvent evaporation, thus inducing improved crystallinity and modulus compared to the higher temperature.

It is also likely that the interaction between temperature and concentration was related to viscosity. Temperature and concentration may have opposite effects on it. Increasing temperature would reduce viscosity, while higher concentration would increase viscosity [55]. Moreover, viscosity was also dependent on temperature, based on Arrhenius-type activation energy [64]. According to the equation, there was a stronger influence of temperature on solution viscosity at higher concentration, as well as a stronger effect of concentration on viscosity at lower temperature. On the other hand, mechanical properties of electrospun fibre were positively correlated to solution concentration [26,35,39]. This further confirms the interaction found in this study. As depicted in Figure 3 and Figure 4, the difference in fibre modulus at different temperatures was more pronounced at higher concentration, whereas the increase in fibre modulus due to concentration was higher at the lower temperature. This suggests that there might be a correlation between solution viscosity and fibre modulus.

The corresponding increase of fibre modulus with respect to flow rate can be seen to be associated with the increasing shear rate. In the case of dry-spinning of PCL, higher shear stress and shear rate improved chain orientation, which corresponded to the higher mechanical properties, until a critical level was reached [39]. Meanwhile, the increase of fibre modulus following higher speed of the rotating collector was also confirmed, in which fibre orientation and point bonding had a role [66].

Applying RSM and optimization procedure, an optimised fibrous PCL biomaterial suitable for synthetic acetabular labrum scaffold has been developed. It had an elastic modulus of 25 MPa with and average fibre diameter of about 1.5 µm. The modulus of the obtained fibre was able to mimic that of acetabular labrum [43], and therefore was potentially suitable as a scaffold to aid labral recovery. Fibril thickness obtained was the smallest possible to be produced using this system, and yet was still capable of accommodating the target value of the elastic modulus. This fibrous morphology can potentially provide a microenvironment that supports cell growth. SEM images (Figure 8) showed that the fibres were reasonably well aligned while providing a sufficient pore volume. Uniformly arranged fibres are reportedly more favourable for cell alignment, proliferation, and extracellular matrix production [33,67]. Meanwhile, pore volume between fibres can facilitate cell infiltration. For the broader context, RSM is also applicable for the development of scaffolds for tissue engineering applications. Once the ideal scaffold parameters have been specified, the various electrospinning parameters can be studied and optimized to achieve a scaffold with the desired properties, in a relatively efficient and straightforward manner.

## 4. Materials and Methods

### 4.1. Electrospinning

Polycaprolactone (Mw 80.000) and acetone (Barnes, Sydney, Australia) were used to make a polymer solution. The PCL solution was prepared by dissolving PCL pellets in acetone overnight. The rotating collector was based an aluminium tube with a gap feature and external covering [68]. A 10 mL syringe with 20 G needle was used to dispense the solution onto a grounded rotating collector. The needle tip was connected to a van der Graaf generator (Serrata, Dural, Australia) to charge the solution. Ejection rate was adjusted using a syringe pump (Injectomat Tiva Agilia, Fresenius Kabi AG, Ba​​d Homburg, Germany). Each sample was produced using approximately 2 mL of PCL solution. Syringe temperature was controlled using a 35 W lamp.

### 4.2. Scanning Electron Microscopy

Scanning Electron Microscopy (SEM–Zeiss EVO, Carl Zeiss AG, Oberkochen, Germany) was used to image the morphological features of the samples. The samples were gold coated and imaged at an operating voltage of 5 kV. Three samples were taken from different sites of a fibre mat obtained from each experimental run. Using SEM images, 30 random measurements of the fibre diameter were performed for each sample using Fiji ImageJ software ( National Institutes of Health, Bethesda, MD, USA), brought in 90 measurements for each experimental run.

### 4.3. Mechanical Testing

Mechanical features of the samples were examined using a tensile test. Rectangular samples of 10 × 80 mm were cut from a membrane produced by each experimental run. Sample thickness was measured using imageJ software from images obtained from optical microscope (Leica microsystem, Wetzlar, Germany). Consecutively, the samples were tensile tested at room temperature, using an Instron 5567 Universal Testing Machine (Norwood, MA, USA) with a 100 N load cell. To avoid slipping, sandpaper was applied to both ends of the specimens. Those samples were then clamped with the length between the clamps set at 60 mm. Strain rate was adjusted to 50 mm/minute and preload was set to 0.2 N. Young’s modulus (E) was calculated from the slope of the stress–strain curve in the linear region.

## 5. Conclusions

This study of electrospinning PCL fibres from PCL/acetone solutions has revealed electrospinning factors that significantly influence the properties of the resulting PCL fibres by applying the RSM technique. For fibre diameter, concentration was the only significant factor. However, concentration, flow rate, rotation speed, and temperature were all determining factors for elastic modulus, as well as the interaction between concentration and temperature. A regression model describing the quantitative relationship between the significant factors and each response was also developed. Using this model, optimum settings to obtain fibres with elastic modulus of 25 MPa at the smallest possible fibre diameter could also be estimated. Independent validation tests showed that the suggested model was applicable and replicable. Based on this result, a follow-up study is now being conducted for the development of an acetabular labrum scaffold based on PCL fibers, including in vitro cell testing.

Development of tissue engineering scaffolds can benefit from the use of the RSM technique, in order to simultaneously investigate multivariate processing factors and multiple responses that often require defining and optimising the outcome quality. With well-defined fabrication parameters and quality characteristics, a step toward scale-up production or even manufacture can be established by this route.

## Figures and Tables

**Table 1:** Parameter settings for the experiment design.

Code	Parameter	Levels		
		−1	0	1
A	Concentration (% *w*/*v*)	10	12.5	15
B	Flow rate (mL/h)	3	4.5	6
C	Distance (cm)	10	12.5	15
D	Temperature (°C)	23	34	45
E	Mandrel rotation speed (RPM)	1000	1500	2000

**Table 2:** Experimental conditions and the response values.

Run	Conc. (% *w*/*v*)	Flow Rate (mL/h)	Distance (cm)	Temp. (°C)	Rotation (RPM)	Average Fibre Ø (µm)	Young’s Modulus (MPa)
1	10	3	10	45	1000	0.903	17.38
2	12.5	4.5	12.5	45	1500	1.742	26.55
3	12.5	4.5	12.5	34	1500	1.436	45.83
4	15	3	15	45	1000	1.741	19.79
5	10	3	15	45	2000	1.810	17.87
6	10	3	10	23	2000	1.239	21.68
7	10	4.5	12.5	34	1500	1.051	24.49
8	12.5	4.5	12.5	34	1500	1.330	27.66
9	12.5	4.5	12.5	34	1500	2.775	57.92
10	15	6	10	45	1000	2.401	31.69
11	12.5	4.5	12.5	23	1500	3.661	63.34
12	10	6	15	23	2000	2.249	55.49
13	12.5	4.5	15	34	1500	2.127	23.55
14	10	6	10	23	1000	2.128	40.70
15	15	3	15	23	2000	2.628	93.08
16	12.5	4.5	12.5	34	2000	2.955	51.81
17	15	3	10	23	1000	2.732	70.22
18	15	4.5	12.5	34	1500	2.726	53.99
19	12.5	4.5	12.5	34	1500	2.571	32.56
20	12.5	4.5	10	34	1500	2.400	44.98
21	12.5	6	12.5	34	1500	2.023	58.49
22	15	6	10	23	2000	2.475	120.02
23	10	6	15	45	1000	2.403	30.26
24	12.5	4.5	12.5	34	1500	2.509	21.02
25	10	6	10	45	2000	3.118	53.15
26	12.5	3	12.5	34	1500	2.134	30.59
27	15	6	15	23	1000	2.562	127.75
28	12.5	4.5	12.5	34	1000	2.171	15.68
29	10	3	15	23	1000	2.100	36.42
30	12.5	4.5	12.5	34	1500	2.181	20.93
31	15	3	10	45	2000	2.665	70.41
32	15	6	15	45	2000	2.478	51.63

**Table 3:** Summary of response surface analysis for fibre diameter.

Terms	Iteration Steps			Terms	Iteration Steps		
	0	1	2		0	1	2
Model	0.416	0.002	0.000	A*C	0.464	-	-
Linear	0.130	0.002	0.000	A*D	0.560	-	-
Square	0.869	-	-	A*E	0.810	-	-
2-way interactions	0.473	-	-	B*C	0.503	-	-
Constant	0.000 *	0.000	0.000	B*D	0.425	-	-
Concentration (A)	0.012 *	0.002	0.000	B*E	0.983	-	-
Flow-rate (B)	0.427	-	-	C*D	0.313	-	-
Distance (C)	0.809	-	-	C*E	0.476	-	-
Temperature (D)	0.548	-	-	D*E	0.112	-	-
Rotation (E)	0.414	-	-	**Model evaluation**			
A*A	0.578	-	-	Lack of Fit	0.628	0.771	0.893
B*B	0.782	-	-	R^2^	67.73%	26.68%	39.12%
C*C	0.916	-	-	R^2^ adjusted	9.06%	24.24%	37.02%
D*D	0.735	-	-	R^2^ predicted	0.00%	16.82%	31.77%
E*E	0.237	-	-	Unusual observation	-	1	-
A*B	0.086	-	-				

* *p* < 0.05 indicates significant variable.

**Table 4:** Summary of response surface analysis for elastic modulus.

Terms	Iteration Steps				
	1	2	3	4	5
	*p*-Value				
Model	0.001 *	0.000 *	0.000 *	0.000 *	0.000 *
Linear	0.000 *	0.000 *	0.000 *	0.000*	0.000 *
Square	0.047 *	-	-	-	-
2-way interactions	0.026 *	0.003 *	0.004 *	0.001 *	0.000 *
Constant	0.000 *	0.000 *	0.000 *	0.000 *	0.000 *
Concentration (A)	0.000 *	0.000 *	0.000 *	0.000 *	0.000 *
Flow-rate (B)	0.003 *	0.006 *	0.009 *	0.001 *	0.000 *
Distance (C)	0.785	-	-	-	-
Temperature (D)	0.000 *	0.000 *	0.000 *	0.000 *	0.000 *
Rotation (E)	0.016 *	0.034 *	0.041 *	0.031 *	0.006 *
A*A	0.649	-	-	-	-
B*B	0.273	-	-	-	-
C*C	0.863	-	-	-	-
D*D	0.253	-	-	-	-
E*E	0.812	-	-	-	-
A*B	0.861	-	-	-	-
A*C	0.884	-	-	-	-
A*D	0.002 *	0.003 *	0.004 *	0.001 *	0.000 *
A*E	0.225	-	-	-	-
B*C	0.521	-	-	-	-
B*D	0.121	-	-	-	-
B*E	0.851	-	-	-	-
C*D	0.039 *	0.075	-	-	-
C*E	0.060	-	-	-	-
D*E	0.132	-	-	-	-
**Model evaluation**					
Lack of Fit	0.855	0.518	0.460	0.800	0.893
R^2^	93.32%	76.03%	72.72%	77.79%	82.09%
R^2^ adjusted	81.18%	70.28%	67.47%	73.16%	78.20%
R^2^ predicted	0.00%	56.21%	54.43%	66.06%	73.24%
Unusual obsv.	-	1	2	1	-

* *p* < 0.05 indicates significant variable.

**Table 5:** Results of confirmation tests.

Run Order	Distance (cm)	Day	Fibre Diameter (µm)	Elastic Modulus (MPa)
1	10	1	1.202	34.46
2	12.5	1	1.127	16.19
3	15	1	1.116	10.06
4	15	2	1.408	23.71
5	12.5	2	1.396	27.92
6	10	2	1.562	30.37
7	12.5	3	1.635	34.70
8	15	3	1.601	33.50
9	10	3	1.457	22.66
Mean ± SD			1.391 ± 0.199	25.95 ± 8.61
Prediction			1.680	25.08

**Figure A1:**
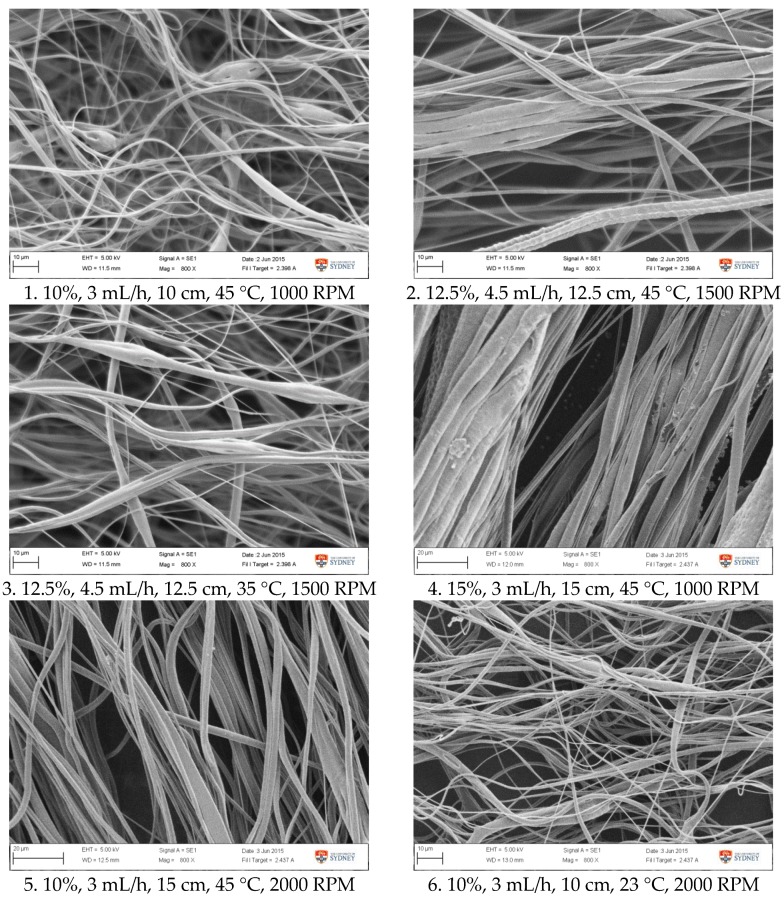

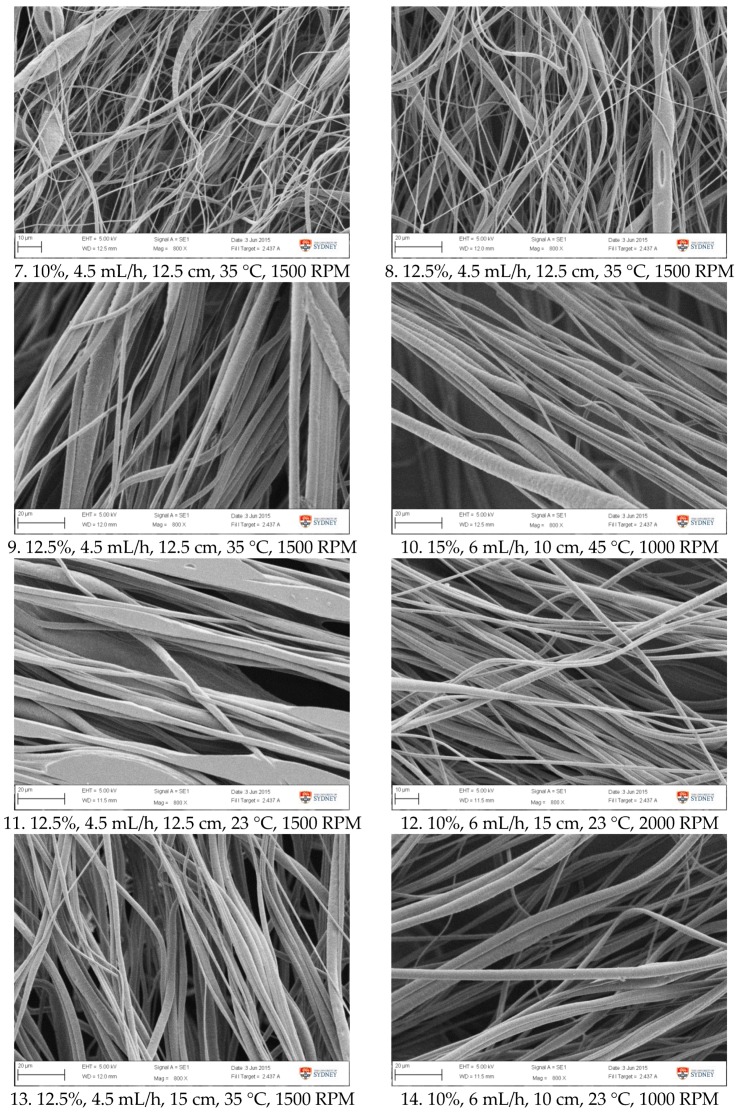

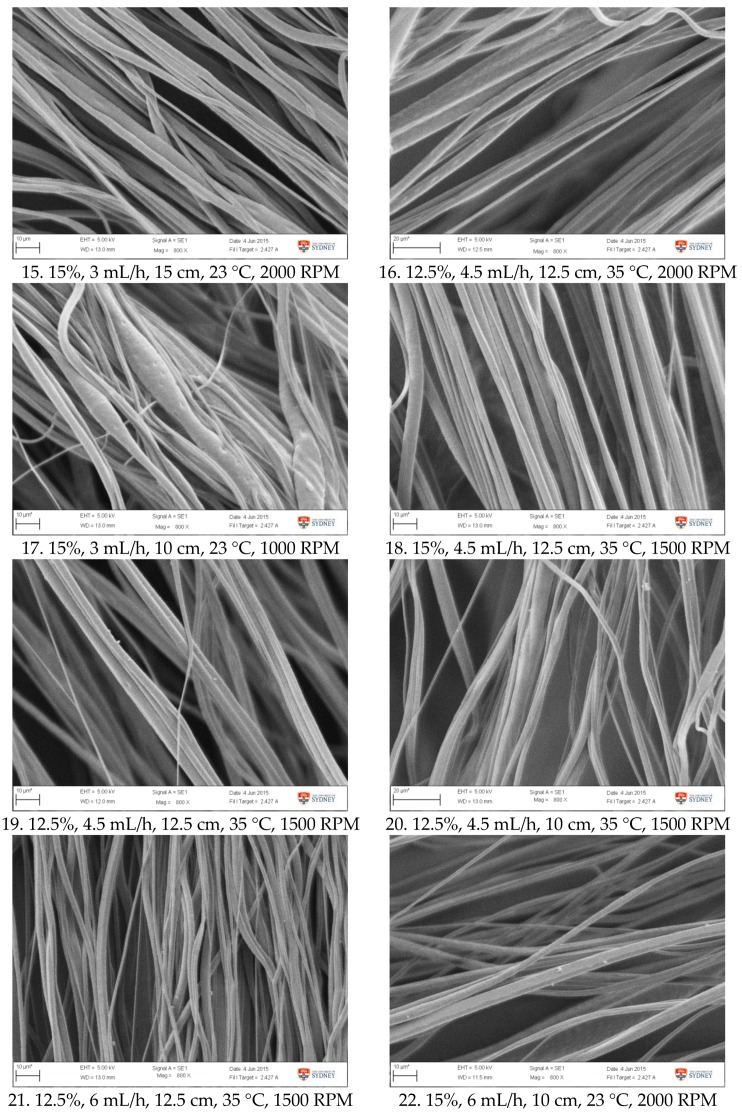

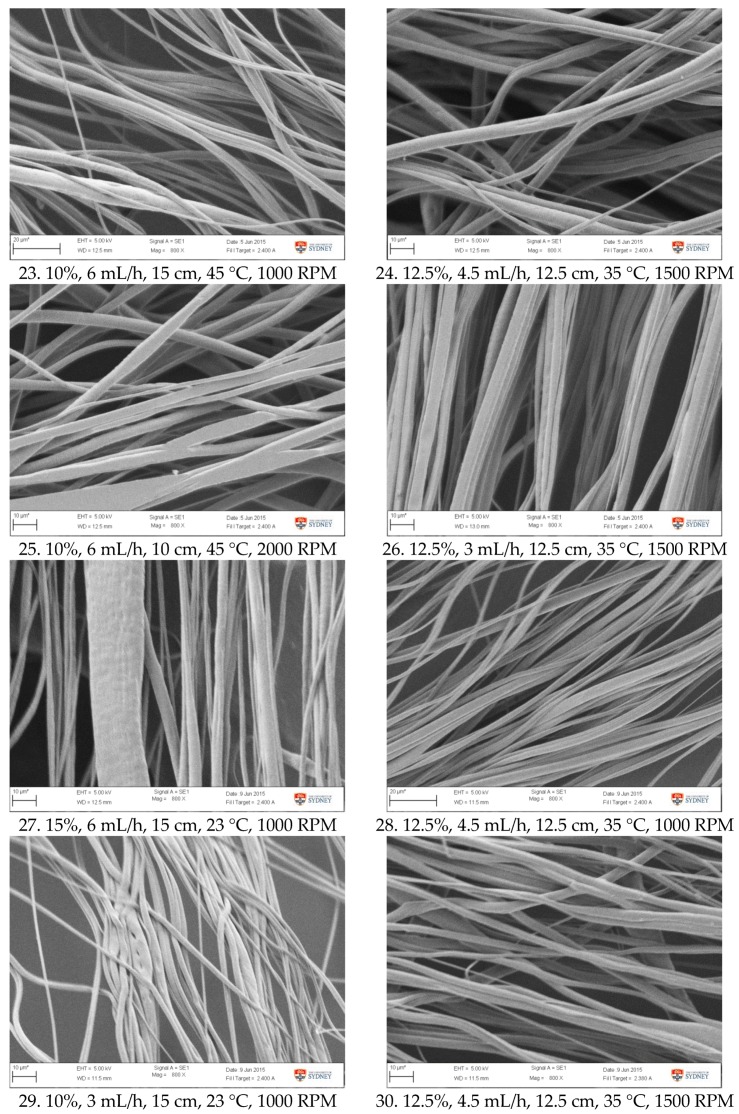

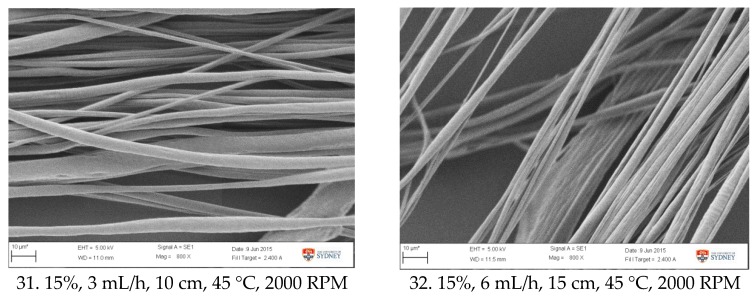
SEM images of fibre morphology from RSM runs.

**Figure A2:**
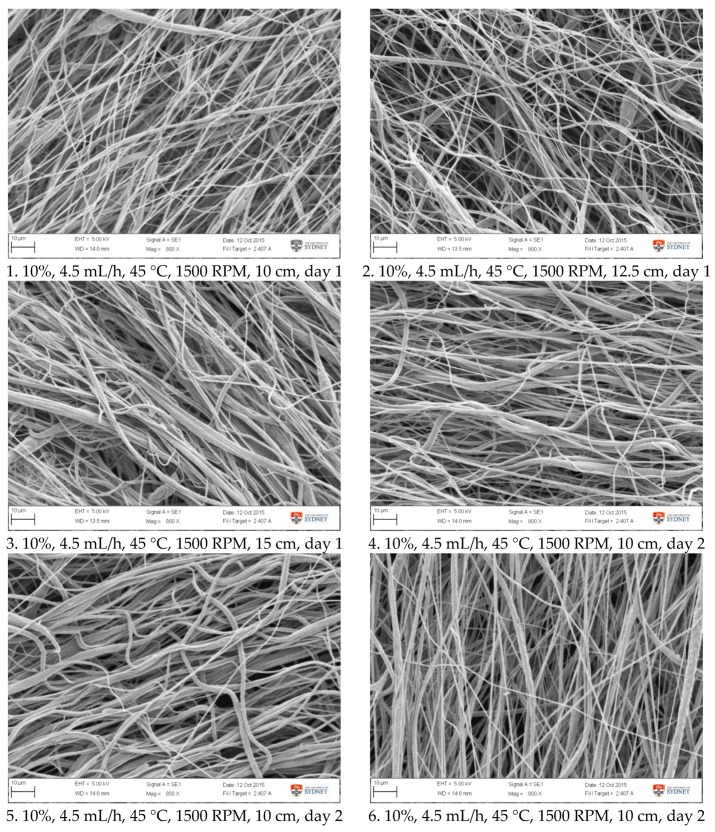

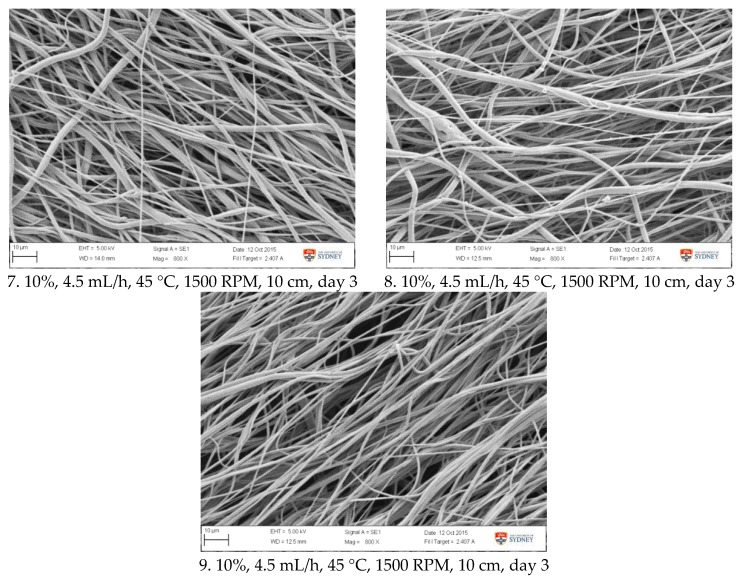
SEM images of fibre morphology from confirmation tests.

## Abbreviations

The following abbreviations are used in this manuscript:

RSM	Response Surface Methodology
PCL	Polycaprolactone
CCD	Central Composite Design
FDA	Food and Drug Administration
RPM	Revolutions per minute
